# Supplementary material for: Finding the equilibrium of organic electrochemical transistors
Source: Nat Commun. 2020 May 20;11:2515. doi: 10.1038/s41467-020-16252-2 (PMC7239912; doi:10.1038/s41467-020-16252-2)
Supplement: Supplementary file 1 — Supplementary Information [file 41467_2020_16252_MOESM1_ESM.pdf]

SUPPLEMENTARY INFORMATION

Finding the Equilibrium of Organic Electrochemical Transistors

Vikash Kaphle[1], Pushpa Raj Paudel[1], Drona Dahal[1], Raj Kishen Radha Krishnan[1] & Björn Lüssem[1,2]

[1]*Department of Physics, Kent State University, Kent, OH, 44242, USA*

[2]*Corresponding Author,* `blussem@kent.edu`

Supplementary Information

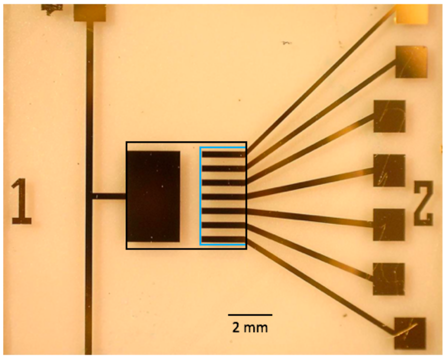

Supplementary Figure 1 Transistor geometry used to study the potential inside the OECT channel. The blue rectangle marks the area, where the channel consisting of PEDOT:PSS is deposited, and the black rectangle represents the part of the device that will be covered by the electrolyte. The left electrode is used as gate, whereas the electrodes on the right are used either as source and drain electrodes or as voltage probes.

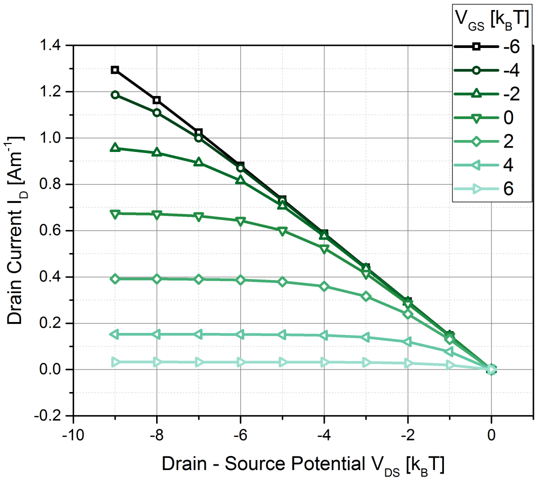

Supplementary Figure 2 Output characteristic. Calculated output characteristic of the OECT shown in Figure 6. Simulation parameters are summarized in Supplementary Table 1.

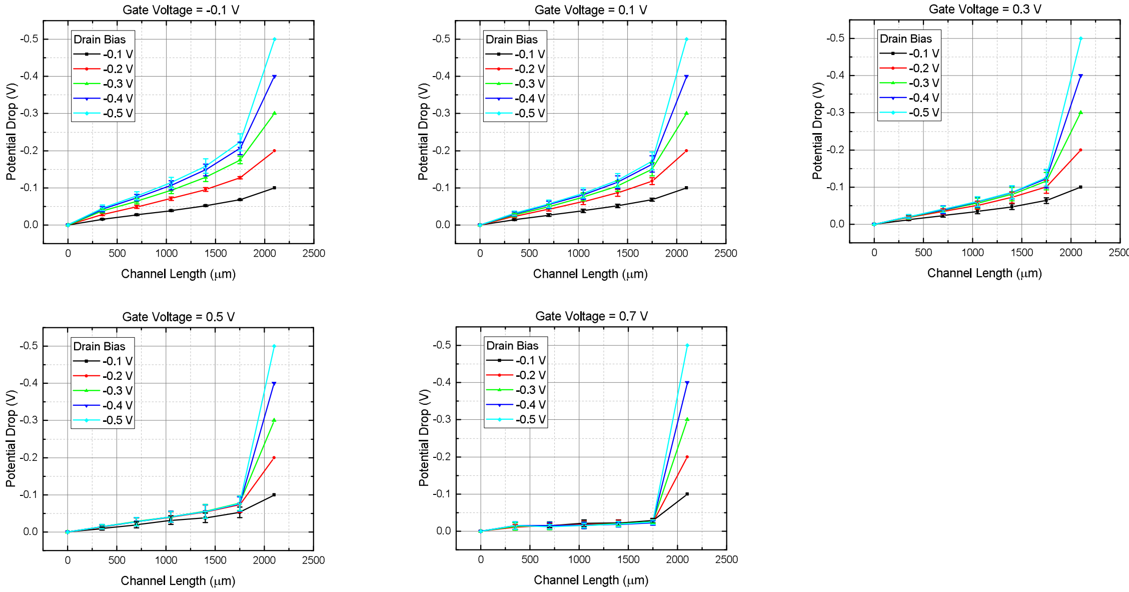

Supplementary Figure 3 Channel potential. Potential inside the transistor channel for all drain and gate potentials.

(a)

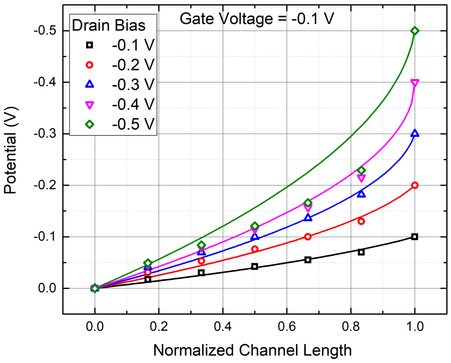

(b)

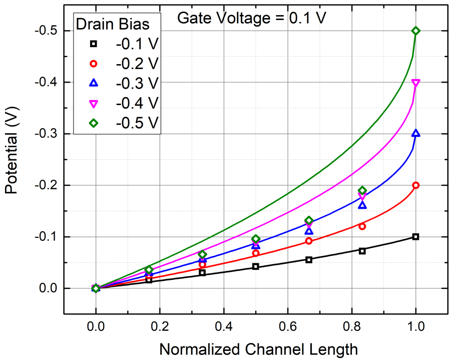

(c)

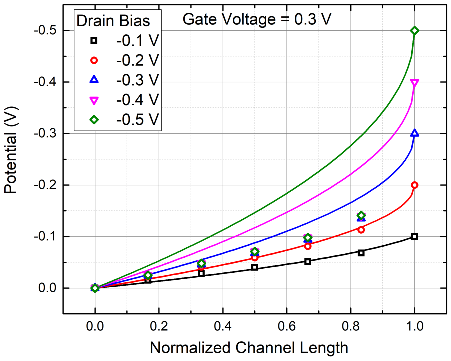

(d)

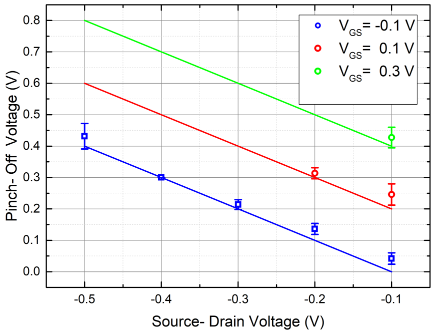

Supplementary Figure 4 Fitting assuming a constant mobility. Channel Potential profile $\Phi(x')$ fitted using Equation 8, i.e. for a constant mobility. **a.** to **c.** show the fit for $V_{\text{GS}} = -0.1V, 0.1V, 0.3V$. **d.** Average pinch-off voltage V_{P} extracted from fitting of the channel potential profile.

(a)

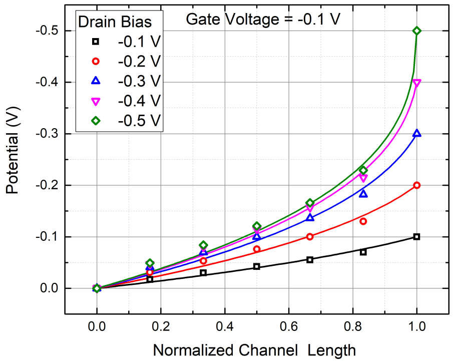

(b)

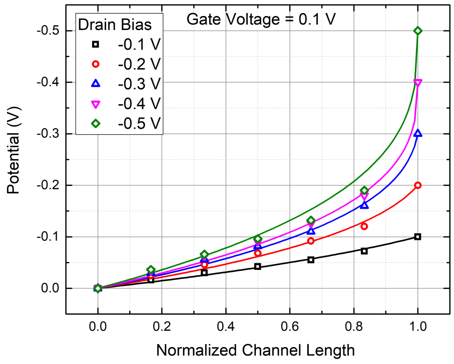

(c)

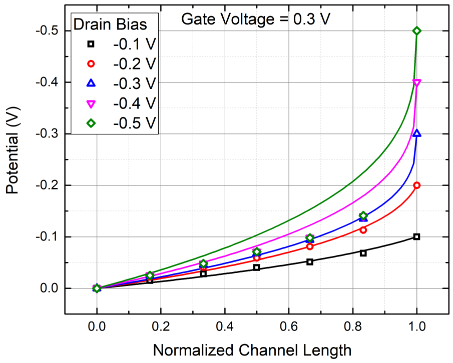

(d)

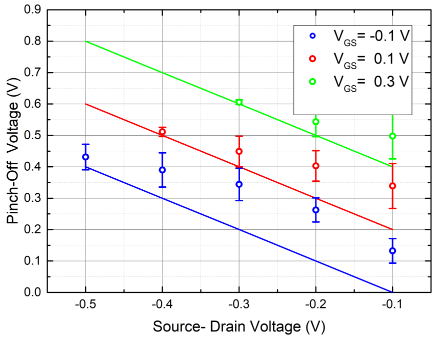

Supplementary Figure 5 Fitting assuming charge carrier dependent mobility. Channel Potential profile $\Phi(x')$ fitted using Equation 9, which takes a dependency of the charge carrier mobility on the hole concentration into account. **a.** to **c.** show the fit for $V_{\text{GS}} = -0.1V, 0.1V, 0.3V$. **d.** Average pinch-off voltage V_{P} extracted from fitting the channel potential profile.

(a) **(b)**

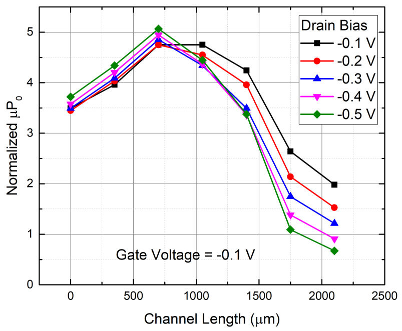

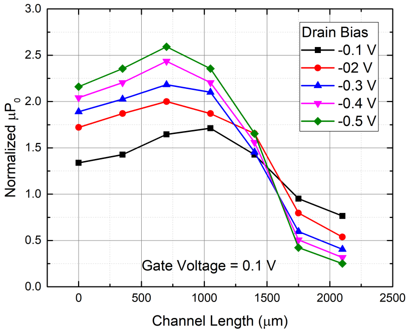

)

(c)

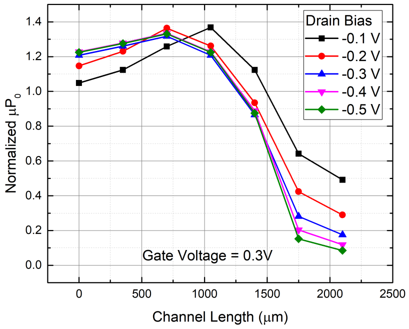

Supplementary Figure 6 Concentration of holes along the channel. Product of hole concentration and hole mobility along the transistor channel for (**a.** to **c.**) $V_{\mathrm{GS}} = -0.1V, 0.1V, 0.3V$.

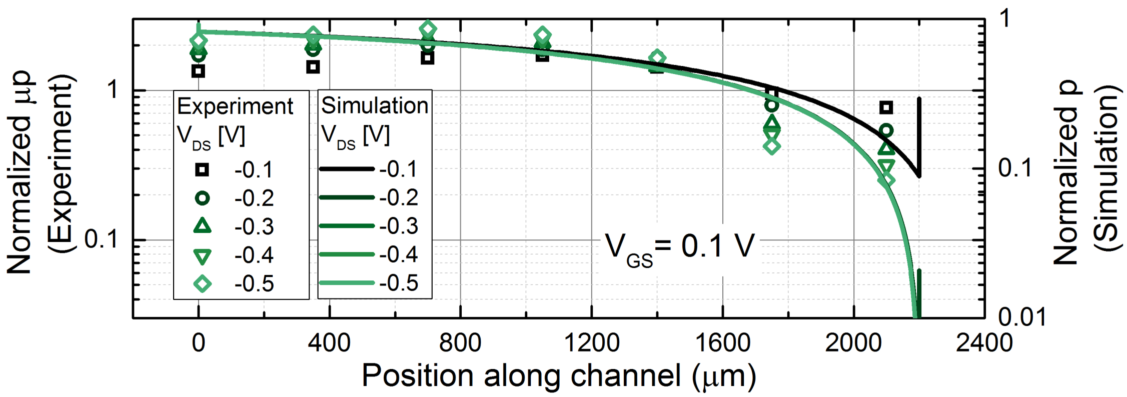

Supplementary Figure 7 Concentration of holes along the channel. Comparison of the normalized product $p(x)\mu(x)$ derived from the experiment (symbols) with the hole concentration $p(x)$ obtained from the 2D model (lines). The parameters listed in Supplementary Table 2 are used for the calculation.

(a) **(b)**

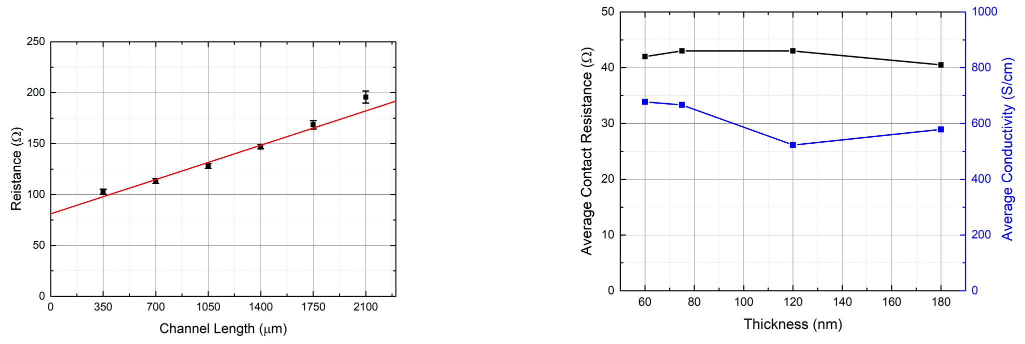

Supplementary Figure 8 Contact resistance. a. Transmission line method to extract contact resistance and conductivity of PEDOT:PSS. **b.** Contact resistance and conductivity of PEDOT:PSS with respect to thickness of the PEDOT:PSS film.

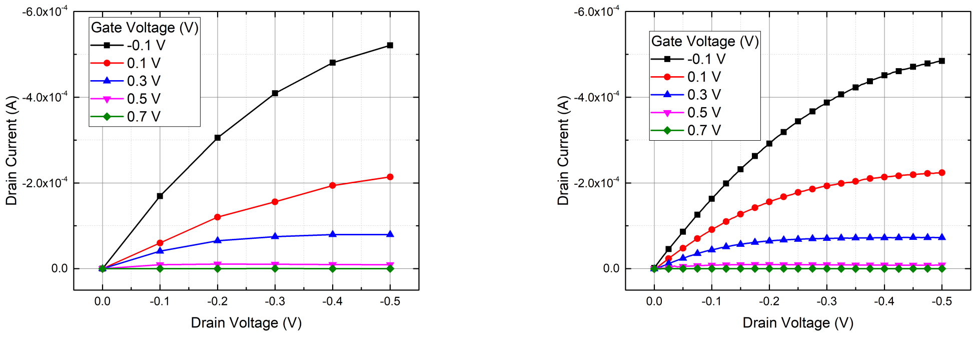

Supplementary Figure 9 Verification of measurement setup. Comparison between the output characteristics (a) extracted current flowing in the channel from the four probe method to measure potential, and (b) from standard drain sweep measurements.

Doping Concentration p_0 $[cm^{-3}]$	Ion Concentration N_0 $[cm^{-3}]$	Hole Mobility μ_p $[cm^2(Vs)^{-1}]$	Cation Mobility $\mu_\text{p,ion}$ $[cm^2(Vs)^{-1}]$
10^{20}	10^{18}	10^{-1}	10^{-4}

Dielectric Constant ϵ	Thickness PEDOT:PSS d_PEDOT $[nm]$	Thickness Electrolyte $d_\text{Electrolyte}$ $[nm]$	Channel Length L $[\mu m]$
3	31.2	10	1.13

Supplementary Table 1 Model parameters used for short channel OECTs. Parameters used for the simulation of OECTs shown in Figure 6.

Channel Length L $[\mu m]$	Doping Conc. p_0 $[cm^{-3}]$	Ion Conc. N_0 $[cm^{-3}]$	Hole Mob. μ_{p} $[\frac{cm^2}{Vs}]$	Contact Resistance R_{C} $[\Omega]$	Cation Mob. $\mu_{\mathrm{p,ion}}$ $[\frac{cm^2}{Vs}]$
350 , 700, 1050<br>1400, 1750, 2100	10^{21}	10^{20}	0.2	40	10^{-4}

Channel Length L $[\mu m]$	Dielectric Const. ϵ	Thick. PEDOT:PSS d_{PEDOT} $[nm]$	Thick. Electrolyte $d_{\mathrm{Electrolyte}}$ $[nm]$	$\Delta\mu$ $[V]$
350 , 700, 1050<br>1400, 1750, 2100	3	120	54	0.4, 0.38, 0.31<br>0.22, 0.15, 0.08

Supplementary Table 2 Model parameters used for long channel OECTs. Parameters used for the simulation of OECTs with varying channel length shown in Figure 8. A small contact resistance was added (cf. Supplementary Figure 8). Furthermore, a difference between the chemical potential of the gate electrode and the source/drain electrode $\Delta\mu$ was assumed.

Supplementary Note 1: Calculation of Channel Potential $\Phi(x)$ and hole concentration $p(x)$ from Bernards model[1] The channel potential inside the channel $\Phi(x)$ can be derived from Equation 4 or the main manuscript, but integrating from source to a point x along the transistor channel instead of integrating from source to drain. This leads to (cf. ref. [2]):

$$\int_0^x j dx = \int_0^{\Phi(x)} -ep(x)\mu(x)d\Phi \tag{1}$$

$$I_\mathrm{D}x = \frac{GLV_\mathrm{P}}{\frac{E_0}{k_\mathrm{B}T}+1}\left\{\left[1-\frac{V_\mathrm{GS}-V_\mathrm{DS}}{V_\mathrm{P}}\right]^{\frac{E_0}{k_\mathrm{B}T}+1} - \left[1-\frac{V_\mathrm{GS}}{V_\mathrm{P}}\right]^{\frac{E_0}{k_\mathrm{B}T}+1}\right\} \tag{2}$$

with $G = p_0 e\mu_0\frac{wd}{L}$. For $\frac{E_0}{k_\mathrm{B}T} = 1$ (i.e. for a constant mobility μ, one obtains

$$\Phi(x') = V_\mathrm{GS} - V_\mathrm{P} + \sqrt{\frac{2I_\mathrm{D}V_\mathrm{P}}{G}x' + (V_\mathrm{P}-V_\mathrm{GS})^2}, \tag{3}$$

where $x' = \frac{x}{L}$. From Equation 6, one finds that $\frac{I_\mathrm{D}V_\mathrm{P}}{G} = \left(V_\mathrm{P} - V_\mathrm{GS} + \frac{V_\mathrm{DS}}{2}\right)V_\mathrm{DS}$, which is used in Supplementary Equation 3 to arrive at Equation 8.

Similarly, one obtains for $\frac{E_0}{k_\mathrm{B}T} = 2$

$$\Phi(x') = V_\mathrm{GS} - V_\mathrm{P} + \sqrt[3]{\frac{3I_\mathrm{D}V_\mathrm{P}^2}{G}x' + (V_\mathrm{P}-V_\mathrm{GS})^3}, \tag{4}$$

which, using Equation 5, can be simplified to Equation 9.

Similarly, one can obtain the hole concentration as a function of x, as already presented by Friedlein et al. [2]

$$\frac{p(x)}{p_0} = \left\{\frac{I_\mathrm{D}\left(\frac{E_0}{k_\mathrm{B}T}+1\right)x}{GLV_\mathrm{P}} + \left(1-\frac{V_\mathrm{GS}}{V_\mathrm{P}}\right)^{\frac{E_0}{k_\mathrm{B}T}+1}\right\}^{\frac{k_\mathrm{B}T}{E_0+k_\mathrm{B}T}} \tag{5}$$

Supplementary Note 2: Determination of hole concentration along the channel. As discussed in the main text, the density of free holes and the charge carrier mobility $p(x)\mu$ can be determined

from Ohm's law $j = -ep(x)\mu\frac{d\Phi(x)}{dx}$ and the experimental measurement of $\Phi(x)$ (cf. Supplementary Figure 6).

Assuming that the variation of the hole mobility μ along the channel is small, the normalized hole concentration $\frac{p(x)}{p_0}$ is proportional to $1 - \frac{p_{\text{ion}}(x)}{p_0}$, i.e. any accumulation of ions at the electrodes will be visible as drop in the hole concentration.

Indeed, it is found that the product of mobility and hole density decreases at the drain contact, which resembles the trend seen in the simulation (Figure 7). In Supplementary Figure 6 an accumulation of holes is visible not only at the drain, but to a lesser extend at the source electrode as well. This observation can be explained by the negative potential difference between source and gate and, consequently, by an accumulation of ions at the source due to this potential difference and consequently a vertical electric field.

Supplementary Note 3: Gradual channel approximation and OECTs. In deriving Equations 5 and 6, the gradual channel approximation was used implicitly. The gradual channel approximation states that the charges in the channel are solely controlled by the vertical electric field E_y generated by the gate potential [3]. In other words, the lateral electric field E_x is assumed to be much smaller than the vertical electric field E_y, which implies as well that $\frac{\partial E_x}{\partial x} \ll \frac{\partial E_y}{\partial y}$. Using the gradual channel approximation, the two dimensional Gauss' law can be reduced to a 1D equivalent

$$\frac{\partial E_x}{\partial x} + \frac{\partial E_y}{\partial y} \approx \frac{\partial E_y}{\partial y} = \frac{\rho(x,y)}{\epsilon}, \tag{6}$$

where $\rho(x, y)$ is the charge density. Supplementary Equation 6 is the justification that the density of free charge carriers inside the channel can be calculated from the difference between the gate potential and the channel potential at position x of the channel only. One obtains for p-type transistors that

$$p(x) = \frac{C_{\text{ox}}}{e} \left(V_{\text{GS}} - \Phi(x) \right), \tag{7}$$

with C_{ox} the gate capacitance.

Equation 1 used to derive Equations 5 and 6 was proposed to resemble Supplementary Equation 7. However, in contrast to standard thin-film theory, Equation 2 enforces that the film is electrically neutral, i.e. $\rho(x, y) = 0$. Therefore, Gauss' law becomes

$$\frac{\partial E_x}{\partial x} + \frac{\partial E_y}{\partial y} = \frac{\rho(x, y)}{\epsilon} = 0, \tag{8}$$

Furthermore, it has been reported that OECTs depend on the thickness of the PEDOT:PSS layer, i.e. the transconductance of the device scales with the volume of the PEDOT:PSS layer. This observation indicates that the electrical potential does not vary along the vertical axis of the device. One obtains

$$\frac{\partial E_x}{\partial x} + \frac{\partial E_y}{\partial y} \approx \frac{\partial E_x}{\partial x} = 0. \tag{9}$$

Therefore, the lateral electric field inside the channel is expected to be constant, i.e. the potential along the channel rises linearly, which is indeed observed in the numerical model.

Overall, it is uncertain if the gradual channel approximation can be applied to OECTs. In the second part of the manuscript, Gauss' law is solved without a-priory invoking the Gradual Channel

Approximation, which makes the conclusion drawn from the improved model more reliable. However, for the first part of the manuscript, Equation 5 and 6 can be seen as first-order approximation of the problem under the assumption of a de-doping process described by Equation 2.

Supplementary Note 4: Drift-Diffusion Simulation Model The drift-diffusion model used here is based on a Finite Difference discretization scheme, which solves Poisson's equation

$$\nabla^2\Phi(x,y) = \frac{e}{\epsilon\epsilon_0}\left(-p(x,y) - p_{\text{ion}}(x,y) + p_0(y) + N_0(y)\right), \tag{10}$$

and the continuity equations for holes and cations in steady state

$$\nabla\left[e(q\mu_{\text{q}}\vec{\mathbf{E}} - D_{\text{q}}\nabla q)\right] = 0 \tag{11}$$

where q can represent holes p or cations p_{ion}. Doping inside the PEDOT:PSS layer is treated by assuming dopant saturation, i.e. all dopants are assumed to be activated. Interactions between cations, holes, and PSS^- is modeled by electrostatic interactions only. All variables $(j, p, n, p_{\text{ion}}, x, y)$ are normalized internally, improving the convergence of the code at high charge carrier concentrations and large electric field observed in the space charge layers.

The three differential equations are solved self-consistently following the Gummel Method. To increase stability, Poisson's equation was adapted as originally proposed by Gummel

$$\nabla^2\Phi(x,y) = -\frac{e}{\epsilon\epsilon_0}\left(\left[\frac{p^k + p^k_{\text{ion}}}{V_{\text{T}}}\left(\Phi^{k+1} - \Phi^k\right)\right] - p^k(x,y) - p^k_{\text{ion}}(x,y) + p_0(y) + N_0(y)\right), \tag{12}$$

where the superscript k and $k+1$ denotes the solution of the kth or $k+1^{th}$ iteration. Please note that the original Poisson Equation is recovered for convergence of the device, i.e. if $\Phi^{k+1} = \Phi^k$.

Boltzmann statistics is assumed throughout the device, which allows to relate the diffusion constant and mobility of holes and ions by the Einstein Equation. Anions in the electrolyte and PSS^- ions are assumed to be stationary.

For results shown in Figure 8, the continuity equation of cations was not solved directly, but the ion concentration was calculated according to Equation 15, which enforces a zero ion current throughout the device. In some cases the results were checked against a full simulation, i.e. a simulation in which the continuity equation for cations is solved explicitly. Furthermore, the results shown in Figure 6 and 7 were obtained by a full simulation of the device.

Supplementary Note 5: Contact Resistance of OECTs. The arrangement of source/drain electrodes as shown in Supplementary Figure 1 allows to determine the contact resistance of PEDOT:PSS even without a gate electrode connected. Here, we process the PEDOT:PSS channel at varying spin coating speed (ranging from 1000 rpm to 4000 rpm), leading to a varying layer thickness (from $180\mu m$ to $80\mu m$ verified by optical profilometry). The resistance of each PEDOT:PSS layer before application of the electrolyte is plotted with respect to the channel length in Supplementary Figure 8a for a 180 nm thick PEDOT:PSS film. Supplementary Figure 8a can be fitted by a linear function; its slope can be used to determine the conductivity of the PEDOT:PSS formulation used here, whereas the intercept represents the combined contact resistance at the source and drain electrode. The contact resistance is found to be approximately equal to $R_{\mathrm{C}} = 80\Omega$.

In Supplementary Figure 8b, the average conductivity and contact resistance are plotted with respect to film thickness. It is found that the contact resistance is independent of the film thick-

ness. Furthermore, the contact resistance represents a significant fraction of the total resistance. In particular for the smallest channel length of $350\ \mu m$, the contact resistance accounts for about $42 \pm 2\Omega$ of the total resistance.

As expected, the conductivity of the films before application of the electrolyte does not depend on the film thickness as well. Overall, the conductivity of the PEDOT:PSS mixed with dodeycelbenzene sulfonic acid (DBSA) and ethylene glycol (EG) is in the range of $611 \pm 73 S/cm$ which is comparable to other literature results[4].

Supplementary Note 6: Verifying the measurement setup. The transistor arrangement as shown in Supplementary Figure 1 can be used to measure the potential inside the transistor channel, if the outer two contacts are used as source and drain contacts and the potential at the electrodes in the middle are used to measure the potential with respect to the source electrode. However, the additional electrodes inside the channel region might change the transistor behavior, for example by changing the distribution of ions inside the channel. To test if this is really the case, we extract the current flowing in the channel at a particular drain and gate bias while measuring the channel potential (shown in Supplementary Figure 9a) and compare it to a standard output characteristic (shown in Supplementary Figure 9b). Both measurements are almost identical, which indicates the transistor behavior is not influenced by the additional sense electrodes within the channel, i.e. the measurement is not falsified.

Supplementary Note 7: Calculation of the hole and cation concentration. The hole concentration plotted in Figure 4a was calculated using Equation 7 of the publication of Friedlein et al.[2]

(cf. Supplementary Equation 5), assuming a constant charge mobility (i.e. $\frac{E_0}{k_{\mathrm{B}}T} = 1$). From the hole concentration, the normalized cation concentration is calculated using Equation 2. Finally, the electric field is calculated from the derivative of the potential along the channel $E_x = -\frac{d\Phi}{dx}$. The potential was calculated using Equation 8.

Supplementary References

1. Bernards, D. & Malliaras, G. Steady-state and transient behavior of organic electrochemical transistors. *Advanced Functional Materials* **17**, 3538 (2007).

2. Friedlein, J. T., Shaheen, S. E., Malliaras, G. G. & McLeod, R. R. Optical measurements revealing nonuniform hole mobility in organic electrochemical transistors. *Advanced Electronic Materials* **1**, 1500189 (2015).

3. Sze, S. & Lee, M. *Semiconductor Devices* (John Wiley & Sons, Inc, 2012).

4. Khodagholy, D. *et al.* High transconductance organic electrochemical transistors. *Nature Communications* **4**, 2133 (2013).
